# Gender differences in response to cold pressor test assessed with velocity-encoded cardiovascular magnetic resonance of the coronary sinus

**DOI:** 10.1186/1532-429X-13-54

**Published:** 2011-09-23

**Authors:** Pierre-Julien Moro, Antonin Flavian, Alexis Jacquier, Frank Kober, Jacques Quilici, Bénédicte Gaborit, Jean-Louis Bonnet, Guy Moulin, Patrick J Cozzone, Monique Bernard

**Affiliations:** 1 Centre de Résonance Magnétique Biologique et Médicale (CRMBM), UMR 6612 CNRS, Université de la Méditerranée, Faculté de Médecine, 27 Bd Jean Moulin, 13385 Marseille cedex 5, France; 2 Service de Cardiologie, Centre Hospitalo-Universitaire La Timone, 264 rue St Pierre, 13385 Marseille cedex 5, France; 3 Service de Radiologie Cardiovasculaire, Centre Hospitalo-Universitaire La Timone, 264 rue St Pierre, 13385 Marseille cedex 5, France

**Keywords:** cold pressor test, coronary sinus flow, endothelium, myocardial blood flow

## Abstract

**Background:**

Gender-specific differences in cardiovascular risk are well known, and current evidence supports an existing role of endothelium in these differences. The purpose of this study was to assess non invasively coronary endothelial function in male and female young volunteers by myocardial blood flow (MBF) measurement using coronary sinus (CS) flow quantification by velocity encoded cine cardiovascular magnetic resonance (CMR) at rest and during cold pressor test (CPT).

**Methods:**

Twenty-four healthy volunteers (12 men, 12 women) underwent CMR in a 3 Tesla MR imager. Coronary sinus flow was measured at rest and during CPT using non breath-hold velocity encoded phase contrast cine-CMR. Myocardial function and morphology were acquired using a cine steady-state free precession sequence.

**Results:**

At baseline, mean MBF was 0.63 ± 0.23 mL·g^-1^·min^-1 ^in men and 0.79 ± 0.21 mL·g^-1^·min^-1 ^in women. During CPT, the rate pressure product in men significantly increased by 49 ± 36% (p < 0.0001) and in women by 52 ± 22% (p < 0.0001). MBF increased significantly in both men and women by 0.22 ± 0.19 mL·g^-1^·min^-1 ^(p = 0.0022) and by 0.73 ± 0.43 mL·g^-1^·min^-1 ^(p = 0.0001), respectively. The increase in MBF was significantly higher in women than in men (p = 0.0012).

**Conclusion:**

CMR coronary sinus flow quantification for measuring myocardial blood flow revealed a higher response of MBF to CPT in women than in men. This finding may reflect gender differences in endothelial-dependent vasodilatation in these young subjects. This non invasive rest/stress protocol may become helpful to study endothelial function in normal physiology and in physiopathology.

## Background

Endothelial dysfunction (ED) is a key element in the development of atherosclerosis and represents one of the earliest manifestations of coronary artery disease [1]. Gender-specific differences in cardiovascular risk have been evidenced by several modalities in human subjects and in animals, and gender-related differences in vascular vasomotion have been documented [2-6]. Differences in the epidemiology of coronary artery disease (CAD) between women and men remain largely unexplained as we are still unable to explain why women are protected towards CAD until older age compared with men [7]. Current evidence supports the role of endothelium in these differences. In particular, the cardiovascular protection in pre-menopausal women is mainly attributed to an enhanced vasodilative capacity of the endothelium [3,4]. Myocardial blood flow (MBF) in men and women has been studied so far mainly using positron emission tomography (PET) in populations of men and women with large ranges of ages [8,9]. A non invasive technique would be useful for measuring myocardial blood flow in any volunteer with normal physiology as well as in patients in a pathophysiological context. Velocity-encoded cine cardiovascular magnetic resonance (VEC-CMR) allows non invasive quantification of blood flow, even in small moving vessels of the cardiovascular system without the use of intravascular catheterization, ionizing radiation, radioactive tracers or gadolinium [10,11]. Van Rossum *et al*. demonstrated the feasibility of VEC-CMR of the coronary sinus measuring global left ventricle (LV) perfusion in healthy volunteers at resting state [12]. The coronary sinus (CS) drains approximately 96% of the total myocardial blood flow making it a practical location to assess global myocardial blood flow. Results of experimental studies using flow probes showed a good correlation between the coronary arterial flow and coronary sinus flow, which indicates that the coronary sinus flow represents LV blood flow [13]. VEC CMR of the coronary sinus for global MBF assessment has also been validated against PET in healthy volunteers [14,15]. Cold pressor test (CPT) allows evaluation of endothelium-dependent coronary vasomotor function mediated by the activation of the sympathetic-adrenergic system, induced by a cold stimulation. Immersion of the extremity (foot or hand) in ice water induces sympathetic stimulation and release of adrenaline and noradrenaline which increases heart rate, arterial blood pressure and myocardial oxygen demand. Sympathetic stimulation facilitates also vasodilation of the resistance arteries by release of NO. This finally leads to increased myocardial blood flow [16,17].

The purpose of this study was to assess non invasively coronary endothelial function in male and female young volunteers by measuring myocardial blood flow (MBF) using coronary sinus flow (CSF) quantification by velocity encoded cine CMR at rest and during cold pressor test (CPT).

## Materials and methods

### Study population

The study was approved by the regional ethics committee (CPP (Comité de Protection des Personnes) Sud Mediterranée). All subjects received both oral and written information and gave written informed consent to their participation in the study. Twenty-four young healthy volunteers (Laboratory staff and medical students) divided into two groups according to gender were examined (12 men, 12 women). Characteristics of the study population are given in Table 1. All subjects were above 18 years of age (age range 18-31 years). All volunteers had a caucasian phenotype. Anthropometric measurements, glucose and lipid profile as well as HOMA-IR (insulin resistance homeostasis model assessment) index were determined. None of the subjects had a clinical history or any evidence of cardiac disease, diabetes, or systemic hypertension. No smokers or obese subjects were included in the study. Each patient was instructed to avoid food and drink containing methyl xanthenes (coffee, chocolate, cola, tea) and alcohol 12 hours prior to the study.

**Table 1:** characteristics of the study population.

	Total *n *= 24	Gender		
		
		Men *n *= 12	Women *n *= 12	p
Age (years)	22 ± 4	23 ± 5	21 ± 3	0.2
BMI (kg/m2)	21 ± 2.5	22.5 ± 2.9	19.8 ± 1.1	0.0124
Waist circumference (cm)	72 ± 9	79 ± 8	66 ± 6	0.0006
Total cholesterol (g/L)	1.7 ± 0.3	1.6 ± 0.3	1.7 ± 0.2	0.1702
HDL cholesterol (g/L)	0.6 ± 0.2	0.5 ± 0.2	0.6 ± 0.2	0.1308
LDL cholesterol (g/L)	0.9 ± 0.2	1 ± 0.2	0.9 ± 0.2	0.9685
Triglycerides (g/L)	0.6 ± 0.2	0.6 ± 0.2	0.5 ± 0.2	0.7463
Fasting glucose (mmol/L)	4.6 ± 0.3	4.7 ± 0.3	4.6 ± 0.4	0.3571
HOMA-IR	1.2 ± 0.7	1.1 ± 0.7	1.3 ± 0.6	0.6301

BMI: body mass index; HDL: high density lipoprotein; LDL: low density lipoprotein; HOMA-IR: insulin-resistance homeostasis model assessment index. p: women vs men, unpaired t test.

### CMR acquisition

CMR was performed on a 3 Tesla scanner (Verio, Siemens, Erlangen, Germany) using a 32-channel phased array coil. Cardiac gating and heart rate measures were achieved using the manufacturer's wireless 3-lead vectocardiogram device. Blood pressure was monitored throughout the protocol using a Maglife system (Schiller). The mean exam time was 30 minutes.

### Cine CMR for LV mass, volumes, and function

Cine-CMR was performed using a retrospectively gated steady-state free precession (SSFP) sequence. Twelve short-axis slices were acquired from apex to base so as to cover the whole LV. The cine-CMR parameters were: repetition time/echo time 62.04/1.24 msec, slice thickness: 6 mm, rectangular field of view 340 × 272 mm^2^, matrix size 256 × 256, GRAPPA factor: 3.

### Coronary sinus flow measurement

CSF measurements were obtained with a non breath-hold flow-encoded fast low angle shot (FLASH) sequence. Following the method described by Schwitter *et al*. [15], the imaging plane was placed perpendicular to the coronary sinus, approximately 2 cm proximal to the entrance of the coronary sinus into the right atrium. As pointed out by Bloch et *al*. [18] as the coronary sinus moves during the coronary cycle we expect different angulations of the slice with respect to the vessel at different cardiac phases. The same authors have shown using phantom measurements that misangulation is not a major error source for angular deviations below 10°. Imaging parameters were: repetition time (acquisition of 1 line of data): 45 msec, echo time: 2.87 msec, Asymmetric echo was used (echo position = 23%), slice thickness: 5.5 mm, field of view: 250 × 250 mm^2^, averages: 11, matrix size: 256 × 256, flow encoding: 70 cm/sec, flip angle: 25°, reconstructed temporal resolution: 40 msec, 4 segments, acquisition time: duration of 262 cardiac cycles, retrospective ECG gating, the number of reference/velocity encoded pairs per cardiac cycle was 4 and the acquisition duration for these 8 gradient echoes was equal to the repetition time (45 msec). The flow encoding was the same for both rest and stress studies and was set to 150 cm/sec. A three-fold GRAPPA acceleration was used to maximize the number of signal averages and to reduce blurring by respiratory motion. Two CSF measurements were accomplished, one at baseline and a second one during CPT.

### Cold pressor test (CPT)

CPT was carried out by immersing the left hand up to the wrist of the volunteer in ice water during the sinus flow measurement (duration: 4 min). Blood pressure and heart rate were recorded at regular intervals before and during CPT. All volunteers tolerated CPT, and measure of MBF was possible in all volunteers.

### CMR image analysis

**Figure 1:**
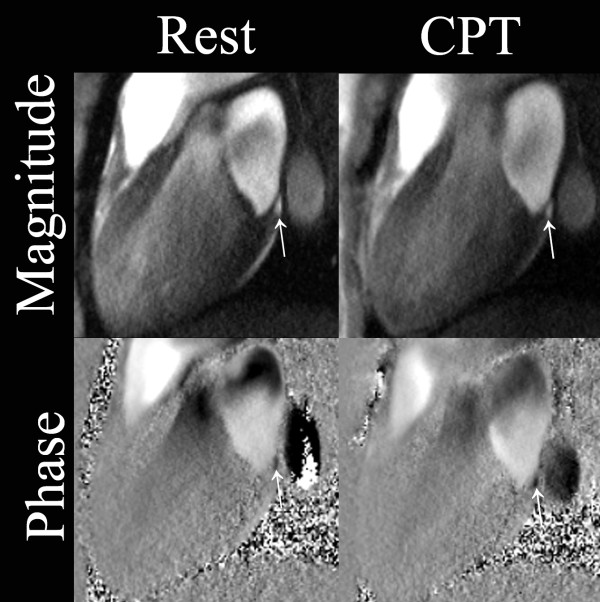
**Coronary sinus (arrows) on magnitude and corresponding phase images**. Long-axis images were obtained at baseline and during CPT using non breath-hold flow-encoded fast low angle shot (FLASH) sequence. Images were acquired at 340 ms in the cardiac cycle.

**Figure 2:**
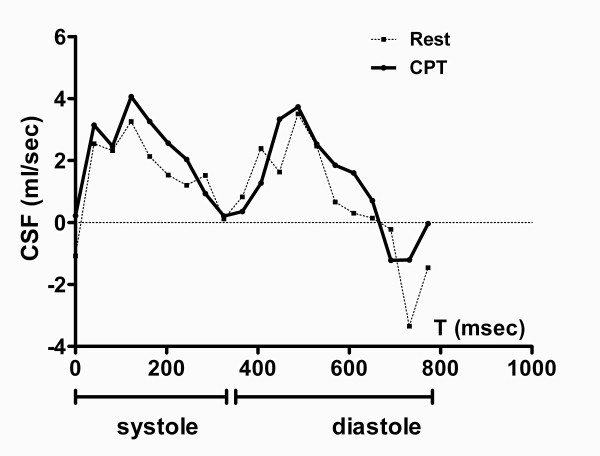
**Coronary sinus volume flow (CSF) during a complete cardiac phase in a healthy volunteer**. CSF at rest and during CPT shows a biphasic pattern: the first peak corresponds to mid systole, the second to early diastole.

Morphological and functional parameters were determined using Siemens Argus 2D software. Endocardial and epicardial contours were drawn in a semi-automated fashion on end-diastolic and end-systolic short-axis images and used to calculate LV volumes, ejection fraction and mass. LV mass and volumes were calculated using Simpson's rule. Coronary sinus flow (CSF) was quantified using Siemens Argus Flow software, the contour of the coronary sinus being manually traced on the magnitude images at each cine frame. The traced region of interest was matched and applied to the corresponding phase image (Figure 1). Average CSF was calculated by integrating the momentary flow values from each cardiac phase (Figure 2) over the entire cardiac cycle and multiplying by the mean heart rate during the acquisition.

### MBF Calculation

MBF was calculated by dividing CSF by LV mass. The rate-pressure product (RPP = systolic blood pressure × heart rate) was calculated as an index of cardiac work and as a measure of the effectiveness of sympathetic stimulation due to peripheral cold exposure. Coronary vascular resistance (CVR = Mean blood pressure/MBF) and finally the endothelium-dependent vasodilatation index (EDVI = MBF after CPT/MBF at rest) were calculated.

### Statistical analysis

All data are expressed as mean ± standard deviation (SD). Differences between myocardial perfusion at baseline and after CPT were analysed with a paired Student's *t *test. Interaction between gender and CPT were analysed by a two-way ANOVA test. The relative MBF difference between rest and during CPT was compared between the two groups using an unpaired Student's t-test. Pearson correlation coefficient was used to assess associations between variables. A p*-*value of less than 0.05 was considered indicating statistically significant difference.

### Inter-observer reproducibility and short term reproducibility

CSF was measured by two observers in 12 subjects. Calculation of MBF was done by both observers using the same data for left ventricular mass, so that the only difference between the observers was due to the drawing of the region of interest in each phase image at rest and during CPT. We compared both rest and CPT measurements in 12 volunteers by a Bland-Altman test. Differences are given as percentage. Short term reproducibility was determined by measuring CSF twice during baseline conditions in a separate group of 5 volunteers and using a Bland-Altman test on MBF measures.

## Results

### Population characteristics

Patient characteristics are shown in table 1. There were no-significant differences in age or in cardiovascular risk factors between the two groups. The body mass index was in the normal range for men, women and the entire population but was significantly lower in women. Waist circumference was also significantly lower in women. Glucose and lipid profiles were normal and not significantly different between the two groups. Both groups did not show insulin resistance as shown by HOMA index.

### LV morphological and functional parameters

Left ventricular (LV) morphological and functional parameters (table 2) were in the normal range in both populations of men and women. There were no significant differences between men and women except for LV mass which was significantly higher in men.

**Table 2:** Cardiac morphology and function.

	Total *n *= 24	Gender		
		
		Men *n *= 12	Women *n *= 12	p
LV mass (g)	99 ± 17	118 ± 19	81 ± 13	10^-5^
EDV (mL)	135 ± 36	149 ± 46	122 ± 15	0.06
ESV (mL)	53 ± 17	58 ± 22	47 ± 8	0.1
LVEF (%)	60 ± 7	61 ± 7	60 ± 7	0.7
SV (mL)	83 ± 22	91 ± 27	75 ± 14	0.08
Qc(L.min^-1^)	5.7 ± 1.3	5.8 ± 1.4	5.6 ± 1.2	0.9

LV left ventricle, EDV end diatolic volume, ESV end systolic volume, LVEF left ventricular ejection fraction, SV stroke volume, Qc cardiac output. p: women vs men, unpaired t test.

### Haemodynamic parameters at rest and after CPT

Haemodynamic parameters at baseline and during CPT are given in table 3. Heart rate, blood pressure and rate pressure product values increased significantly and similarly in men and women although there were differences in basal and CPT heart rate and RPP.

**Table 3:** Haemodynamic parameters.

	Total *n *= 24	Gender		
		
		Men *n *= 12	Women *n *= 12	p
HR (min^-1^)				
Rest	66 ± 11	60 ± 7	73 ± 12	0.003
CPT	89 ± 11*	80 ± 12*	99 ± 17*	0.005
Rel. change (%)	35 ± 18	34 ± 21	37 ± 16	0.75
BP (mmHg)				
Rest				
Systolic	114 ± 8	114 ± 7	114 ± 10	0.89
Mean	84 ± 9	83 ± 9	85 ± 9	0.77
Diastolic	71 ± 9	71 ± 8	71 ± 9	0.95
CPT				
Systolic	129 ± 11§	132 ± 13§	126 ± 8§	0.20
Mean	99 ± 11§	100 ± 13§	98 ± 9*	0.64
Diastolic	85 ± 12*	86 ± 13§	84 ± 11*	0.60
Rel. change (%)				
Systolic	13 ± 10	16 ± 11	11 ± 8	0.26
Mean	19 ± 14	20 ± 17	17 ± 11	0.60
Diastolic	20 ± 15	19 ± 12	22 ± 18	0.58
RPP (mmHg·min^-1^)				
Rest	7738 ± 1790	7136 ± 1497	8340 ± 1914	0.10
CPT	11404 ± 2497*	10341 ± 2028*	12466 ± 2541*	0.03
Rel. change (%)	50 ± 29	49 ± 36	52 ± 22	0.83

HR heart rate, BP blood pressure, CPT cold pressure test, RPP rate pressure product. p, women vs men, unpaired t test. *p < .0001 vs. rest, §p < .002 vs. rest, paired t test.

### MBF response to CPT

**Figure 3:**
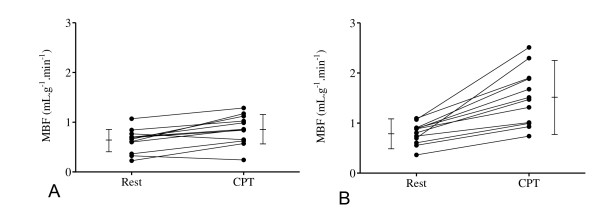
**Individual comparison of myocardial blood flow (MBF) at rest and during CPT for each healthy volunteer**. A) men B) women.

MBF increase in each volunteer is shown in Figure 3. MBF and CVR responses to CPT are shown in table 4. MBF increased significantly in men (by 43 ± 49% (p = 0.0022)) and more than twice as much in women (by 92 ± 50% (p = 0.0001)). The difference of flow between cold pressor testing and baseline (ΔMBF) was statistically significant *(*p = 0.001) for men and women (table 4 and Figure 3). In two men, no MBF increase was observed. Under cold pressor test, RPP increased similarly in both men (by 49 ± 36%) and women (by 52 ± 22%). Coronary vascular resistance diminished significantly in women (38 ± 13% (p < 0.0001)) but not in men (10 ± 32%). EDVI was 1.38 ± 0.64 for men and 1.92 ± 0.51 for women. Among the total population significant associations of ΔMBF were found with ΔHR (pearson r = 0.88, p = 0.0002), with ΔRPP (pearson r = 0.50, p = 0.01) and with waist circumference (pearson r = -0.46, p = 0.03). No significant association was found with body mass index, LV mass and blood biochemical variables (glycemia, triglycerides, total, LDL and HDL cholesterol, HOMA index of insulin resistance).

**Table 4:** Comparison of results in men and women in respect of myocardial blood flow (MBF), coronary vascular resistance (CVR) and endothelium-dependent vasodilation index (EDVI) at rest and in response to CPT.

	Total *n *= 24	Gender		
		
		Men *n *= 12	Women *n *= 12	p
MBF (mL·g^-1^·min^-1^)				
Rest	0.71 ± 0.23	0.63 ± 0.23	0.79 ± 0.21	0.08
CPT	1.19 ± 0.55	0.85 ± 0.29*	1.52 ± 0.56*	0.001
ΔMBF from rest (mL·g^-1^·min^-1^)	0.47 ± 0.42	0.22 ± 0.19	0.73 ± 0.43	0.001
CVR (mmHg·mL^-1^·g·min)				
Rest	139 ± 74	162 ± 93	115 ± 40	0.1
CPT	106 ± 75	139 ± 92	71 ± 27*	0.01
EDVI	1.67 ± 0.55	1.38 ± 0.64	1.92 ± 0.51	0.03

p women vs men, unpaired t test. *p < 0.005 vs rest, paired t test.

### Reproducibility

**Figure 4:**
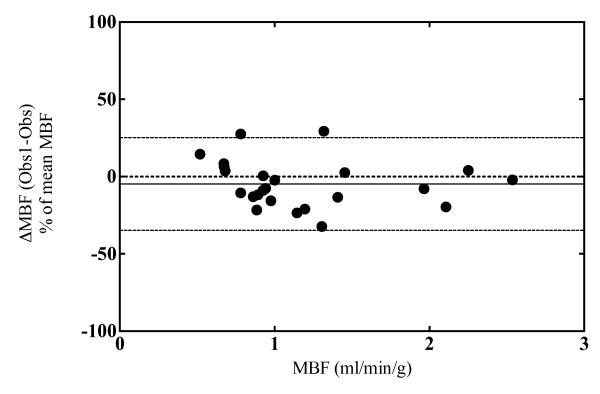
**Bland-Altman plot assessing inter-observer variability obtained by two independent researchers in a subgroup of 12 subjects during rest and CPT**. Bias was found to be -4.7% (-34.7% to +25.2%). Dashed line shows the bias; dotted lines correspond to the 95% limits of agreement.

Inter-observer variability bias assessed in a subgroup of 12 subjects at rest and during CPT was found to be -4.7% (-34.7% to +25.2%) (Figure 4). Short-term variability for baseline flow measurement was 3.5% (-30.3% to 37.2%).

## Discussion

In this study, we used CSF measurement by CMR in combination with CPT to assess the influence of gender on CPT-induced changes in MBF, exploring a young population of men and women thanks to the non invasiveness of the method. Non-invasive quantification of coronary endothelial function using myocardial blood flow at the venous coronary sinus site combined with CPT was shown sensitive enough to measure global myocardial blood flow changes in healthy volunteers. As an internal validation, we found that post-processing was reproducible between different operators.

In this study, MBF values (0.71 ± 0.23 mL·g^-1^·min^-1^) obtained at rest were in accordance with the results of previous CMR studies using VEC-CMR reporting values in a range between 0.52 ± 0.21 and 0.74 ± 0.23 mL·g^-1^·min^-1 ^[14,15,18-22]. VEC-CMR was previously validated against PET with values ranging from 0.57 ± 0.15 mL·g^-1^·min^-1 ^to 0.91 ± 0.15 mL·g^-1^·min^-1 ^[10,14,15,23]. VEC-CMR was also validated against invasive quantification of myocardial blood flow using flow probes [13]. As VEC-CMR measure had a 3 to 4 min duration, one concern was the time course of the vasodilatory response to CPT. Kiviniemi *et al*. [24], using transthoracic echocardiography, were able to perform several measurements of epicardial coronary artery diameter and coronary flow velocity during CPT. They showed that vasodilatation was stable during the duration of the CPT [24]. In a subsequent study they used averaged measurements of epicardial coronary artery diameter and coronary flow velocity during the 3 min duration of CPT [25]. Consequently, the duration of the CMR sequence used in the present study is consistent with the time course of the CPT vasodilatory effect. Short term variability was comparable to previous studies [15]. For interobserver variability, bias was comparable to previous studies [15,18] but the range was wider than described by Schwitter *et al*. [15].

To date, CMR perfusion measurements combined with CPT were reported from two other studies, one of which also employed coronary sinus flow measurements and CPT [22], but aimed at a larger population featuring various cardiac pathologies. The other study used first-pass perfusion CMR to assess MBF changes while inducing coronary spasm by CPT in a younger population, but without assessment of gender differences [26].

Pharmacologic stress tests using adenosine or dipyridamole measure the combined effect of vascular smooth muscle relaxation and endothelium mediated vasodilatory function and are considered to reflect the total coronary vasodilating capacity [10], although the endothelium-dependent response is only partial. In contrast, CPT is a specific stimulus of the coronary vascular endothelium [16,17]. It is an interesting tool for studying normal physiology, but also from a diagnostic point of view as endothelial dysfunction has been considered as the "ultimate risk factor" for cardiovascular diseases [27,28]. Endothelial dysfunction contributes to a wide range of pathophysiological processes including hypertension, coronary heart disease, stroke, diabetes and atherosclerosis. Detection of endothelial dysfunction could therefore allow clinicians to identify patients at risk [27] and to stratify their cardiovascular risk [29]. Diminished MBF response to CPT has indeed been shown to be associated with endothelial dysfunction [1].

Even though normal endothelial function is not a computable value, Schindler *et al*. [29] showed a higher incidence of cardiovascular events in patients with impaired endothelial function. The authors, by studying sympathetic stimulation by PET during CPT, demonstrated that a negative or an impaired response to CPT was associated with a higher incidence of cardiovascular events [29]. Other studies assessing coronary endothelial vasoreactivity in patients with cardiovascular disease have also concluded that an altered endothelial function is a major prognostic factor for adverse cardiovascular events [30,31]. Beyond diagnosis, the assessment of ED is of great importance in the choice of therapeutics [23]. An available non invasive method to quantify ED would therefore provide a relevant tool to study the response to treatment and to monitor therapy in diseases that modify endothelial function, such as diabetes, hypertension, and dyslipidemia.

As expected, the increase in MBF obtained with CPT is lower compared to the effect observed with the pharmacological agents. We found CPT-induced MBF values of 1.19 ± 0.55 mL·g^-1^·min^-1 ^representing a 68 ± 55% increase compared with baseline values. This is in agreement with previously published data combining PET and CPT, in which MBF values under CPT were found between 0.88 ± 0.27 mL·g^-1^·min^-1 ^[1] and 1.26 ± 0.07 mL·g^-1^·min^-1 ^[32]. PET MBF changes between rest and CPT reported in the literature range from 33% [29] to 83% [23] in healthy volunteers. As a comparison, an increase from 0.64 ± 0.9 to 1.59 ± 0.79 mL·g^-1^·min^-1 ^was reported using VEC-CMR and dipyridamole as a stress agent [29].

Previous studies have shown significant differences in baseline MBF between male and female subjects [8,33]. In this study, there was a trend to higher baseline MBF in women compared to men, although without statistical significance. Interestingly, our results show a higher increase in MBF during CPT in women. As shown by others [8] we found significant associations between ΔMBF and ΔHR and ΔRPP. However increases in HR and RPP from baseline to cold pressor testing were comparable between men and women suggesting that the different response to CPT is not related to differences in heart rate or cardiac work increases. Besides gender, within the young population studied here, differences in MBF response were not related to differences in age, BMI, LV mass or blood biochemical parameters. Higher MBF response to endothelial stimulus in young women is consistent with experimental evidence of differences in endothelial-dependent vascular reactivity between males and pre-menopausal females [3-5]. A number of studies have documented the interaction of estrogens with endothelial function [7] both in clinical studies and experimental models. Estrogens and their receptors play a key role in endothelium-dependent maintenance of vascular tone [3-5]. Estrogens decrease systemic vascular resistance, improve coronary and peripheral endothelial function and prevent coronary artery spasm in women with and without coronary atherosclerosis [7]. Estrogens also modulate relaxation through the endothelium-derived hyperpolarizing factor (EDHF), by inducing vasodilator prostanoids (PGE2, PGI2), and by inhibiting the production of endothelin-1 [7]. In addition, chronic estrogen treatment enhances endothelium-dependent vasodilation in large peripheral arteries of postmenopausal women [34,35].

Furthermore endothelial progenitor cells (EPCs) play an important role in vascular response. A recent study based on 210 healthy subjects demonstrated higher steady-state levels of EPC in fertile women than in men, while they were not different between postmenopausal women and age-matched men. EPCs are mobilized cyclically in fertile women in synchrony with the level of circulating 17 beta-estradiol, and they could represent an important mechanism of protection for premenopausal women [7,36]. Cellular intrinsic sex related differences within the endothelial cells have also been shown to contribute to differences between males and females [6]. In summary, differences in endothelial function between male and female may at least partially explain the lower incidence of cardiovascular disease in pre-menopausal women. Other factors that modulate or alter autonomic cardiac activity, may potentially influence sex differences, such as inflammation, increased pain sensitivity to cold, and psychological disorders (e.g. depression). A lower central sympathetic neural output to the periphery and a lower sympathetic vasoconstrictor drive in healthy women has been described compared to healthy men [37]. Furthermore less visceral abdominal fat, may also explain the difference in vasomotion between men and women [38] as a matter of fact we found that waist circumference was lower in women than in men and that ΔMBF was negatively correlated to waist circumference. In an earlier study using PET, Prior *et al*. [8] showed no differences in MBF response to CPT between men and women in normal volunteers, but the range of ages was rather large in both men (19-75 years) and women (24-66 years). However, to our knowledge this is the first study directly assessing gender MBF differences in a homogeneous population of healthy young volunteers.

In this study, we have chosen to perform the measure of MBF during free breathing, and it should be noted that improvements of the flow imaging technique can potentially be obtained by using respiratory gating with a navigator echo as reference. This should reduce breath motion blurring and therefore improve the true spatial resolution of the technique at the expense of measurement time. The use of a 3 Tesla system combined with a 32-channel thoracic array is likely to have contributed to make the image quality sufficient for this study. Parallel imaging with GRAPPA acceleration has allowed for a high number of signal averages leading to reduced respiratory artifacts.

## Conclusion

CMR coronary sinus flow quantification as a measure of myocardial blood flow without contrast agent revealed higher vascular response in young women than in young men, which may reflect well-documented gender differences in endothelial-dependent vasodilatation. Better understanding of these processes may improve the clinical management of CAD in women. Due to the small sample size these results should be confirmed on a larger population before being generalized and other aspects such as the role of hormonal status, inflammation biomarkers, pain sensitivity and psychological evaluation that could contribute to sex differences will need also to be investigated.

CSF measurements combined with CPT are non-invasive and may be helpful for assessing variations of endothelial function in normal physiology and in pathologies, in which changes in endothelial function occur early. It might therefore be a candidate as a diagnostic tool for cardiovascular alterations in diabetes, coronary artery disease, hypertension or atherosclerosis.

## Competing interests

The authors declare that they have no competing interests.

## Authors' contributions

PJM and AF; acquisition and analysis of data, draft of the manuscript. AJ, FK, MB; conception and design, acquisition, analysis and interpretation of data, draft and revision of the manuscript. BG; analysis of data. JQ, JLB, GM and PJC, revision of the manuscript. All authors have read and approved the final manuscript.
